# Comprehensive Assessment of Secreted Immuno-Modulatory Cytokines by Serum-Differentiated and Stem-like Glioblastoma Cells Reveals Distinct Differences between Glioblastoma Phenotypes

**DOI:** 10.3390/ijms232214164

**Published:** 2022-11-16

**Authors:** Laverne D. Robilliard, Jane Yu, Akshata Anchan, Graeme Finlay, Catherine E. Angel, E Scott Graham

**Affiliations:** 1 School of Medical Sciences, Department of Molecular Medicine and Pathology, Faculty of Medical and Health Sciences, University of Auckland, Auckland 1023, New Zealand; 2 Centre for Brain Research, University of Auckland, Auckland 1023, New Zealand; 3 Auckland Cancer Society Research Centre, University of Auckland, Auckland 1023, New Zealand; 4 School of Biological Sciences, Faculty of Science, University of Auckland, Auckland 1010, New Zealand

**Keywords:** glioblastoma, cytokines, stem-like, immuno-modulatory, leukocyte recruitment, serum-differentiated, GBM, human

## Abstract

Glioblastoma is refractory to therapy and presents a significant oncological challenge. Promising immunotherapies have not shown the promise observed in other aggressive cancers. The reasons for this include the highly immuno-suppressive tumour microenvironment controlled by the glioblastoma cells and heterogeneous phenotype of the glioblastoma cells. Here, we wanted to better understand which glioblastoma phenotypes produced the regulatory cytokines, particularly those that are implicated in shaping the immune microenvironment. In this study, we employed nanoString analysis of the glioblastoma transcriptome, and proteomic analysis (proteome profiler arrays and cytokine profiling) of secreted cytokines by different glioblastoma phenotypes. These phenotypes were cultured to reflect a spectrum of glioblastoma cells present in tumours, by culturing an enhanced stem-like phenotype of glioblastoma cells or a more differentiated phenotype following culture with serum. Extensive secretome profiling reveals that there is considerable heterogeneity in secretion patterns between serum-derived and glioblastoma stem-like cells, as well as between individuals. Generally, however, the serum-derived phenotypes appear to be the primary producers of cytokines associated with immune cell recruitment into the tumour microenvironment. Therefore, these glioblastoma cells have considerable importance in shaping the immune landscape in glioblastoma and represent a valuable therapeutic target that should not be ignored.

## 1. Introduction

Glioblastoma (GBM) is the most common and most deadly brain tumour, with median survival times of 15 months following diagnosis. Inevitable recurrence and no effective therapies lead to less than 5% of patients alive after five years of disease progression. Most notably, glioblastoma has seen limited benefits from immunological intervention, despite promising outcomes in other cancer types such as melanoma, lung cancer and prostate cancer [1]. These therapeutics include the promising checkpoint inhibitors, CAR-T therapy, oncolytic virus delivery, dendritic cell vaccination and other personalised approaches [2]. As glioblastoma are regarded as immunologically ‘cold’ tumours, it is not surprising that many immune-targeted therapies fail to meet clinical outcomes. However, the value of understanding the immunological microenvironment should not be understated.

GBM microenvironments are intrinsically heterogeneous with tumour cells existing on a continuous spectrum of stemness to a more differentiated phenotype. The cellular state of glioblastoma arises due to innate plasticity dependent on the microenvironment [3]. The roles of both stem-like and differentiated cellular states have been shown to influence the immune landscape of glioblastoma tumours, primarily through integrin receptor binding. For example, IGFBP10 secreted by a differentiated model of GBM contributes to macrophage infiltration, while secretion of the integrin ligand, periostin, by SOX2^+^OLIG2^+^ GBM stem-like cells (gCSCs) promotes M2 macrophage recruitment specifically [4,5]. Acknowledging the inherent differences in immune regulation by different cellular states is necessary to develop appropriate immunotherapies.

**Figure 1:**
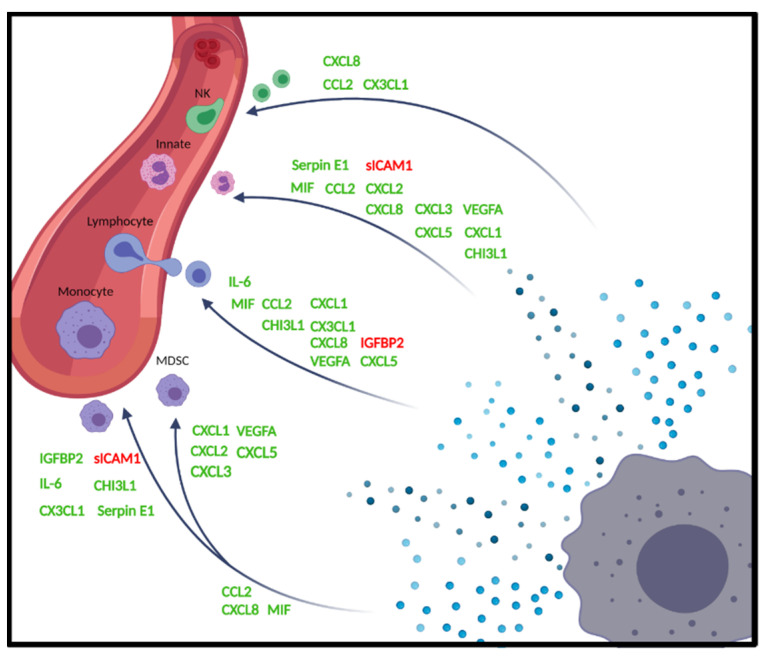
Secreted proteins associated with immune recruitment into tumour microenvironments. Glioblastoma cells secreted a milieu of soluble proteins into the tumour microenvironment. These soluble molecules comprise chemokines capable of acting on the blood–brain barrier to promote peripheral immune cell recruitment, cytokines that activate or suppress immune cell function and other immune modulators responsible for shaping tumour immune responses. Green = positive recruitment, red = negative recruitment.

The complexity of the glioblastoma immune microenvironment likely impacts clinical outcome during disease progression [6]. While glioblastoma has been shown to contain various immune populations, these infiltrates frequently display markers of immunosuppression. Glioblastoma-associated macrophages (GAMs) are the most abundant cell type in these infiltrates, with single-cell level technology mass cytometry (CyTOF) revealing that macrophage populations make up 59% of the CD45+ immune cells within GBM microenvironments [7]. GAMs are actively recruited into the tumour core through CCL2, SDF-1, M-CSF and GM-CSF signalling, and undergo further polarisation to promote a pro-tumorigenic environment [8,9]. Hierarchical cluster analysis of GAM populations in glioblastoma tumours demonstrates that there is substantial intra-tumoral diversity within this immune population. In certain clusters, GAMs displayed heightened expression of PD-L1, further evincing the active role GAMs play in promoting immune suppression [7]. Similarly, T-cells within GBM microenvironments demonstrate a suppressed phenotype. Fu et al. (2020) have also used single cell CyTOF analysis to reveal an extensively suppressed phenotype of both CD4+ and CD8+ T cells as defined by increased PD-1, TIM-3 and LAG-3 expression compared to peripheral cells [7]. Interestingly, a population of CD8+CD28-FoxP3+ Tregs was also identified in 60% of glioblastoma patients. Despite representing only 2.08% of all infiltrating T-cells, CD8+ Tregs shift antigen presenting cells (APCs) into an anti-inflammatory phenotype by down-regulating APC costimulatory molecule expression. They also exert cytotoxic effects on CD4+ T cells [10,11]. Natural killer cells (NK) are integral to appropriate anti-tumour immune responses. Their presence within tumour microenvironments should, ideally, reflect effective tumour cytolysis. In glioblastoma, the small number of NK cells that do infiltrate the tumour express low levels of interferon-γ and are no longer cytolytic, and also have decreased expression of activating receptors [7,12]. The indisputably immune-suppressed GBM microenvironment poses significant therapeutic hurdles. Mechanistically, the milieu of secreted molecules (see Table 1 and Figure 1 summary) by distinct tumour cell subsets likely contributes significantly to the ‘cold’ nature of these tumours.

Here, we have defined a set of secretory immune modulators that are highly expressed in glioblastoma cell cultures and have examined the implications of an enhanced stem-like phenotype. Furthermore, using the CIBERSORT algorithm, we sought to understand the association between specific secretory molecules (as shown in Figure 1 and Table 1) and both the presence and phenotype of immune-infiltrating cells in a cohort of glioblastoma patients. This study provides an appreciation of the influence of secreted cytokines, chemokines and immune modulators on the recruitment and phenotype of immune populations in the GBM microenvironment. Specifically, the study demonstrates important differences in the secretome of serum-derived and gCSC glioblastoma cellular states, thus supporting the necessity to study each of these glioblastoma phenotypes in the pathophysiology of GBM.

## 2. Results

### 2.1. Gene Expression Profiling Reveals a Distinct Subset of Immune Modulators That Are Highly Upregulated in Glioblastoma Cells

**Figure 2:**
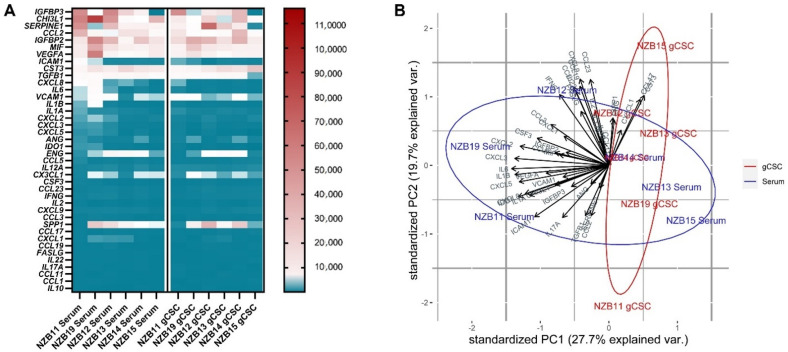
Secreted protein gene expression profiles comparing serum and gCSC glioblastoma cells. (**A**) NanoString median mRNA count in serum-derived and gCSC primary glioblastoma cell lines. (**B**) Principal component analysis of log10 transformed nanoString intensities. The first and second principal components show 27.7% and 19.7%, respectively.

The glioblastoma microenvironment comprises of a specific subset of immune infiltrates, dominated by macrophages. The comparatively low levels of other immune cell types suggest that a specific set of secreted molecules is present in the tumour microenvironment, biased towards monocyte/macrophage recruitment. NanoString mRNA quantification of a comprehensive list of cytokines, chemokines and immune modulators identified a specific set of genes that is highly expressed in cell pellets prepared from six glioblastoma cell lines and their stem-like cell counterparts (Figure 2A).

The pleiotropic cytokine, IL-6, is implicated in the recruitment of monocytes and lymphocytes, and IL-6 mRNA is abundantly present in all glioblastoma lines investigated (Figure S1). Of the nine cytokines analysed, IL-1Β was the only additional gene significantly expressed (Figure S1). Cytokines are the backbone of immunological regulation. Importantly, their presence, or lack thereof, is indicative of the phenotypes immune infiltrates acquired once entering tissue environments. The selective expression of IL-6 and IL-1 mRNA is intriguing as both are implicated in the induction of myeloid-derived suppressor cells in the tumour microenvironment. The distinct lack of other cytokine expression infers mechanisms at play responsible for effectively limiting the exposure of immune infiltrates to appropriate signalling within the GBM microenvironment.

The chemokine genes *MIF*, *CCL2*, *CXCL8*, *CX3CL1* and *CXCL2* were all highly expressed by the six glioblastoma primary lines (Figure S2). MIF (monocyte inhibitory factor) is positively associated with the recruitment of monocytes, T cells, neutrophils, and monocyte-derived suppressor cells (MDSC). Similarly, CCL2, CXCL8 and CXC3L1 are involved in the recruitment of leukocytes, including NK cells. Conversely, CXCL2 is restricted to the recruitment of MDSCs and innate immune cells. While these chemokines are classically regarded to recruit immune cells into tissue, they also exert pleiotropic effects in the regulation of immune subsets. MIF is reported to be involved in inhibiting NK function, while CXCL2 is associated with an increase in MDSC activity [53,54].

Of the non-classical immune modulators investigated, genes for CHI1L3, SERPINE1, VEGFA, IGFBP2, IGFBP3, SPP1, CST3, TGFB1, ENG, ICAM1, VCAM1 and ANG were abundantly expressed (Figure S3). CHI1L3, Serpin E1 and IGFBP2 are all associated with an increase in monocyte abundance in tumour tissue. High levels of VEGFA are reported to increase the movement of inhibitory MDSCs into tissue, while IGFBP2 is involved in suppressing the influx of T-cells (as highlighted in Figure 1 and Table 1).

An important consideration regarding the gene expression profile of secreted immune modulators is patient variability. Here, Principal Component Analysis (PCA) adequately represents the extent of variability that exists between glioblastoma cultures (Figure 2B). The low variance seen in PC1 and PC2 indicates that there is significant variation between the differing patient cell lines that is unable to be explained by the expression profiles of immune modulators.

### 2.2. Secretome Analysis of Immune Modulatory Molecules Successfully Delineates Stem-like Populations from Serum Cells

**Figure 3:**
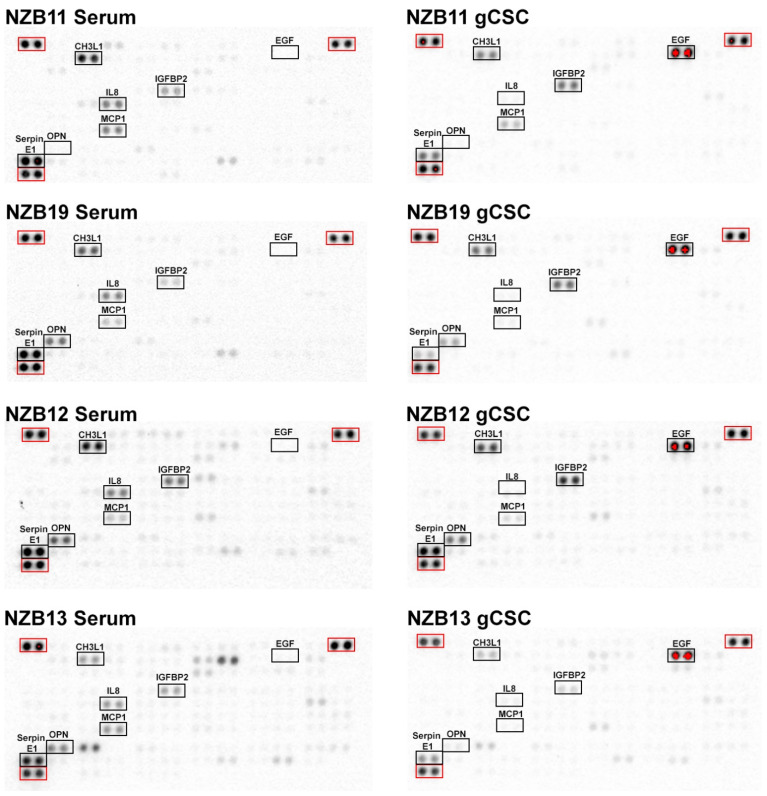
Proteome Profiler Human XL cytokine arrays of NZB glioblastoma conditioned medium. NZB11, NZB19, NZB12 and NZB13 glioblastoma cell 48 h conditioned medium. Arrays were imaged equivalently by chemiluminescence. Shown is each array 10 s before reference spot saturation (red).

**Figure 4:**
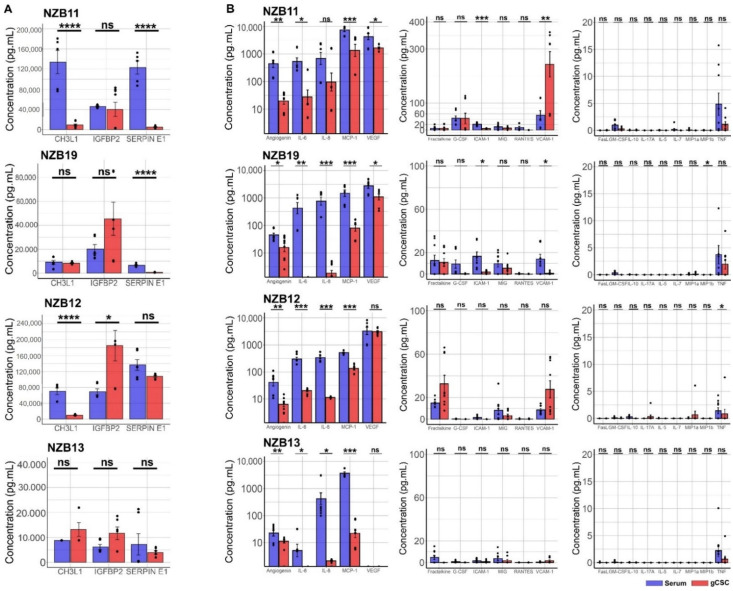
Concentrations of secreted immune-modulators in 4 glioblastoma cell lines. (**A**) Luminex based analysis of CHI3L1, IGFBP2 and Serpin E1 concentrations in NZB11, 12, 13, and 19 cell lines cultured in serum or as stem-like cells. (**B**) Cytometric bead array analysis of selected cytokines and chemokines concentrations in NZB11, 12, 13 and 19 cell lines cultured in serum or as stem-like cells. The results of three independent experiments are shown. Unpaired students *t*-test analysis was carried out. *p*-value = 0.05 (*), 0.01 (**), 0.001 (***), 0.0001(****), and ns means not significant.

**Figure 5:**
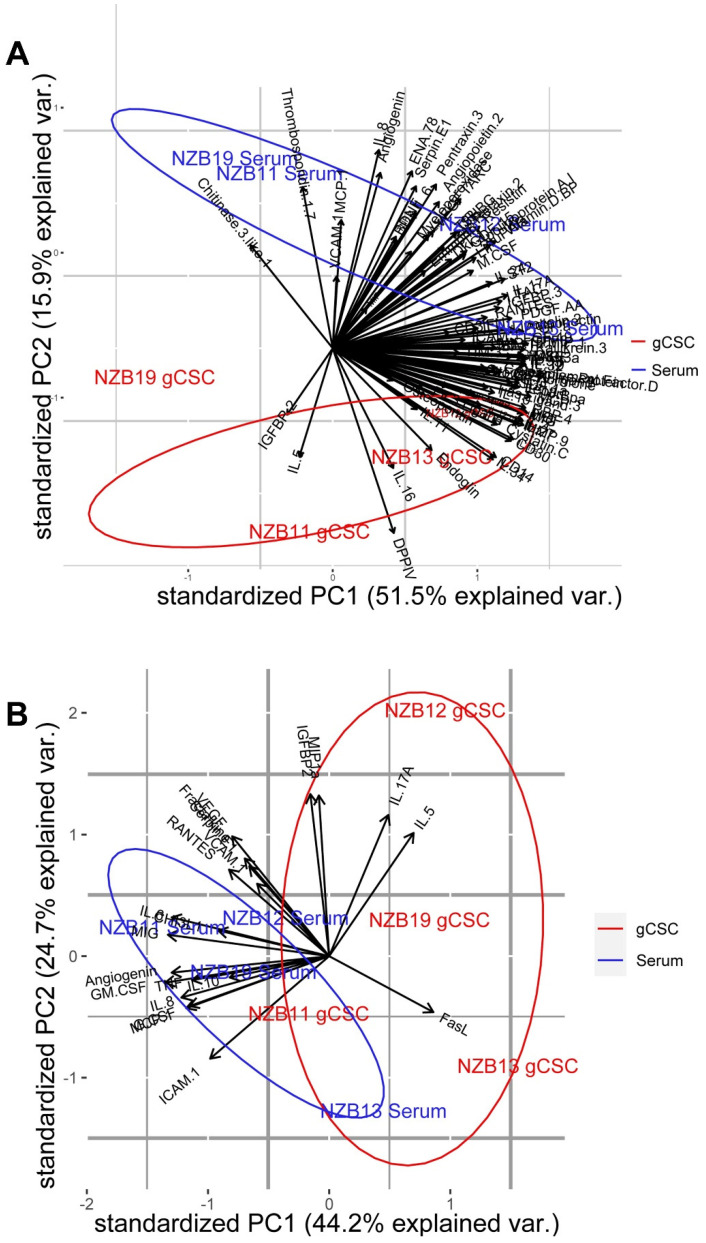
Principal Component analysis of key cytokines, chemokines and immune modulators in 4 GBM cell lines cultured as serum or stem-like cells. Principal component analysis was carried out on (**A**) mean log10 transformed proteome profiler intensities and (**B**) mean log10 cytometric bead array and luminex concentrations. The first and second principal components show 51.5% and 15.9% (Proteome Profiler), and 44.2% and 24.7% (CBA and Luminex) of the total variance, respectively.

**Figure 6:**
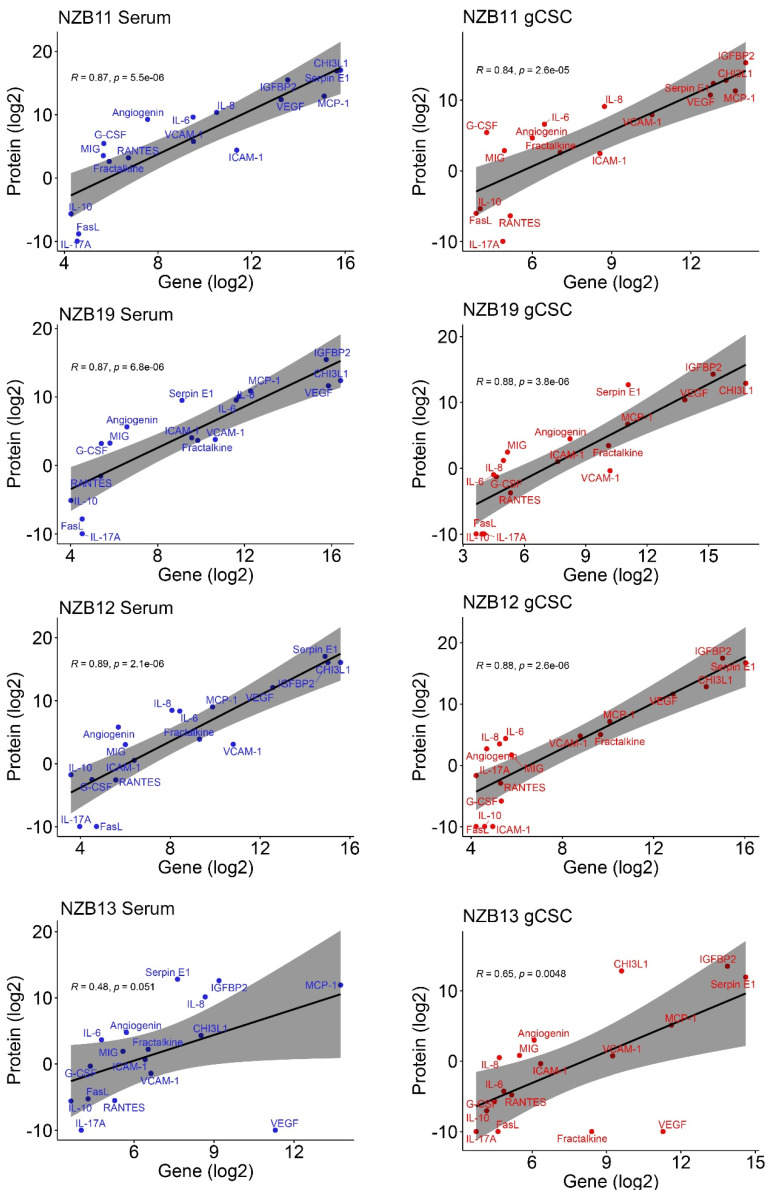
Secreted protein vs. gene expression correlation. Cytometric bead array and Luminex secreted protein concentrations, and NanoString mRNA count comparison in NZB11, NZB19, NZB12 and NZB13 serum and stem-like cell cultures. Coefficient of correlation (*R*) and related *p* values are shown (*p*). Red wording denotes gCSC data where blue denotes serum cultures.

To determine the concentrations and relative differences between serum and stem-cell like cultures, six glioblastoma cell lines were screened for the expression of secreted proteins using Human Cytokine proteome profilers, cytometric bead arrays and Luminex assays (Figure 3 and Figure 4). Cytokine proteome profiling provides a broad-based screen for 105 targeted soluble proteins. NZB11, NZB12, NZB13, NZB14, NZB15 and NZB19 serum cells were compared with their gCSC counterparts for the presence of key soluble proteins (Figure 3, Figure 4 and Figure 5). Corroborating the NanoString mRNA analysis, the Human Cytokine Arrays detected strong signals for CHI3L1, IGFBP2, CXCL8 (IL-8), CCL2 (MCP1) and Serpin E1 in all six cell lines (Figure 6).

An important consideration for this study is the comparison between the glioblastoma secretome profiles of cells derived in serum compared to the stem-cell counterparts. Glioblastoma stem-cells are an important subset of the tumour cell population; however, little is known about their role in sculpting the immune landscape. Proteome profiling was able to detect elevated levels of IGFBP2 in gCSC cultures compared to the serum cultures. This is in contrast to the overall trend observed: that gCSCs tend to produce lower levels of CHI3L1, CXCL8, CCL2 and Serpin E1 (Figure 3). Importantly, this observation is confirmed by Principal Component Analysis (PCA) (Figure 5), where serum cultures secreted a greater abundance of the majority of the secreted proteins detected.

To validate the Human Cytokine proteome profiler analysis, Cytometric Bead Array (CBA) and Luminex-based assays were employed. These assays utilise bead-based technology to bind soluble proteins found in conditioned medium and quantify their subsequent concentrations based on recombinant protein standard curves. The primary objective of these assays was to measure the concentrations of important immune modulators defined by NanoString and proteome profiler screens. Furthermore, the sensitivity of the assays also allowed for the direct comparison of soluble protein concentrations in conditioned media from serum-derived cells and their gCSC counterparts (Figure 4).

Luminex and CBA quantification confirmed high levels of CHI3L1 (0–6705 pg/mL), IGFBP2 (309.5–3454 pg/mL), Serpin E1 (328.4–6833 pg/mL), Angiogenin (27.27–614.3 pg/mL), IL-6 (12.57–788.1 pg/mL), CXCL8 (IL-8) (359.9–1317 pg/mL), CCL2 (MCP-1) (522.1–7786 pg/mL), and VEGF (0–5370 pg/mL) in all serum-derived glioblastoma primary lines (Figure 4). Unlike mRNA analysis, which showed relatively equivalent gene expression between serum-derived and gCSC cultures, secretome analysis reveals that gCSC cultures significantly down-regulate the production of numerous soluble proteins, and up-regulate others. There is significant down-regulation of CHI3L1, Serpin E1, Angiogenin, CXCL8 (IL-8), IL-6, CCL2 (MCP-1) and VEGF secretion. However, it appears that soluble VCAM-1 (sVCAM-1) and IGFBP2 are up-regulated by some gCSC cultures (Figure 4). While the up-regulation observed is not consistent across all lines investigated, it alludes to a potential role these molecules may play in altering the immune environment influenced by the glioblastoma cell niche.

An important observation to note, aligning with nanoString data, is the astounding lack of secreted cytokines and common chemokines such as RANTES, G-CSF, GM-CSF, MIP-1α, IL-10, and IL-17A (Figure 4 and Figure 6). The data here indicates that the stem-like cellular compartment demonstrates decreased capability to recruit anti-tumour immune populations, while retaining the ability to engage other immune populations through sVCAM-1 and IGFBP2 production. PCA analysis validates that IGFBP2 up-regulation is mostly associated with an increased stem-like phenotype, while simultaneously verifying that serum cultures are the primary producers of most soluble molecules (Figure 5).

### 2.3. TCGA GBM RNA-seq Immune Subset Deconvolution Correlates Highly Secreted Immune-Modulators with Immune Infiltration and Activation Status

**Figure 7:**
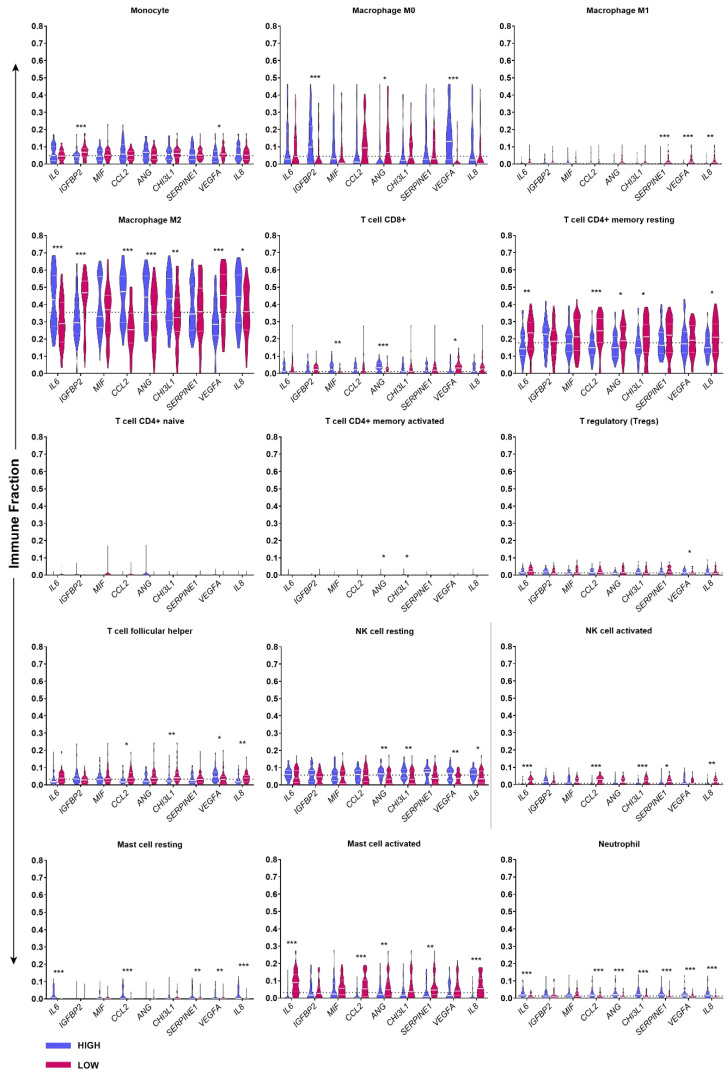
Violin plots of TCGA GBM RNA-seq CIBERSORT immune subset deconvolution. CIBERSORT immune subset deconvolution was applied to 160 publicly available GBM RNA-seq dataset. Data were separated into quartiles to define high expressing samples (Q1) (n = 40) from low expressing samples (Q4) (n = 40) for *IL6*, *IGFBP2*, *MIF*, *ANG*, *CHI3L1*, *SERPINE1*, *VEGFA*, and *IL8*. Data were processed using TIMER 2.0 software to output relative fractions of leukocytes based on the validated CIBERSORT leukocyte gene signature matrix. Shown are the relative fractions of 15 leukocyte subsets comparing high and low expressing samples across eight genes of interest. Violin plots represent the median (solid line) and quartiles (dotted lines). The dotted line across each graph represents the median relative scored in the whole dataset (n = 160). Mann–Whitney *U* test analysis was carried out. *p*-value = 0.05 (*), 0.01 (**), 0.001 (***).

Utilising data publicly available through the Cancer Genome Atlas (TCGA) the influence of key soluble proteins on immune infiltration in patient glioblastoma resections was determined. RNA-seq data from the resected tissue was analysed by the CIBERSORT algorithm. CIBERSORT deconvolution based on a 547-leukocyte gene signature was sensitive enough to reveal significant differences in immune fractions depending on the mRNA expression of *IL6*, *IGFBP2*, *MIF*, *ANG*, *CHI3L1*, *SERPINE1*, *VEGFA*, and *IL-8* (Figure 7). To note, MDSCs were not included in the immune subset deconvolution. CIBERSORT deconvolution reaffirms that the primary immune population within glioblastoma samples are macrophages, specifically M2 macrophages. T-cell CD4+ memory resting cells make up the second most abundant immune population. CD8+ T-cells, follicular helper T cells, resting NKs, monocytes, MO macrophages and activated mast cells equally contribute to the remaining populations present in the cohort of GBM tumours (Figure 7).

While CD4+ memory resting T cells contribute most T-cells within the tumour microenvironment (TME), their presence is highly influenced by CCL2, ANG, CHI3L1, IL-6 and IL-8. Low expression of these genes positively correlates with an increase in the memory resting population. Comparatively, elevated levels of ANG, MIF and CHI3L1 gene expression appear to be associated with an increase in CD8+ T cells. VEGF, a molecule secreted unanimously by all lines investigated, appears to be significantly correlated with a decrease in CD8+ T cells (Figure 7). As an appropriate cytotoxic T cell response is an essential component of anti-tumour responses, the negative effect of VEGF on infiltration is intriguing (Figure 7).

Like cytotoxic CD8+ T cells, natural killer cells are essential for anti-tumour responses. NKs recognise aberrant cells and induce cytolysis through receptor engagement and perforin/granzyme release. CIBERSORT deconvolution suggests a clear and systematic mechanism that might explain inhibition of NK function. High mRNA expression of ANG, VEGFA, CHI31, CCL2, IL-6, SERPINE1 and IL-8 collectively promote a resting/inactive NK phenotype and prevent the accumulation of activated NK cells (Figure 7). The same trend is seen in mast cell populations whereby IL-6, Serpin E1, IL-8 and CCL2 are associated with an increase in resting phenotypes and a decrease in activated phenotypes (Figure 7).

The presence of M2 macrophages within the TME is classically associated with pro-tumorigenic activity [55]. High expression of IL6, CHI3L1, ANG, IL-8 and CCL2 predictably correlate with increased M2 macrophage presence. However, high levels of VEGFA, IGFBP2 and SERPINE1 appear to negatively correlate with M2 and M1 phenotypes and promote a shift to an M0 phenotype. The findings indicate that the presence of VEGFA, IGFBP2 and SERPINE1 prevents polarisation of monocytes within the TME, restricting macrophages to a resting state [56].

Ideally, the tumour immune microenvironment would be characterised by high infiltration of cytotoxic T-lymphocytes, M1-polarised macrophages, high levels of antigen presentation, and low T-reg and MDSC numbers [57]. The presence of cytokines and chemokines such as IL-6 and CCL2 is known to shift this ‘ideal’ immune microenvironment to one that is immunosuppressed, similar to the immune profile described by the TCGA analysis shown here.

Gene Set Enrichment Analysis was performed to determine whether genes within the defined GO terms are correlated with either the Q1 (high gene expression) or Q4 phenotype (low gene expression). The GSEA algorithm sorts genome-wide data into an organised ranked gene list, with genes highly correlated with the Q1 phenotype at the top of the list. The algorithm then sets out to determine whether genes within the defined GO terms are found at the top, bottom or randomly throughout the ranked gene list. The enrichment score is determined based on each gene within the defined GO terms position in the ranked gene list. Enrichment plots and Normalised Enrichment Scores for the GO terms macrophage activation, T cell activation, NK activation, macrophage migration, T cell migration, and T cell proliferation are shown (Figure S4). Samples with high IL-6, CCL2, CHI3L1, ANG, IL-8 and SERPINE1 expression are enriched for gene ontologies involved in macrophage, T-cell and natural killer cell activation and migration.

## 3. Discussion

The glioblastoma immune microenvironment comprises a complex interactome of resident and peripheral immune subsets, many of which are immunosuppressive. Effective therapeutic strategies rely on functional immune clearance of tumour cells in coordination with treatment. Currently, approved treatments for glioblastoma (surgery, radiation, temozolomide and dexamethasone) all likely alter the immune composition and function within the tumour microenvironment [2]. While it is widely accepted that monocytes and macrophages constitute most of the immune population within glioblastoma, T cells, natural killer cells and other peripheral subsets undeniably contribute to glioblastoma pathogenesis [58].

Both the myeloid and lymphoid immune compartments significantly participate in glioblastoma immunosuppression. Extensive genotypic profiling of infiltrating monocytes by Gabrusiewicz et al. (2016) provides evidence of the considerable complexity of GAMs, for example [59]. GAMs have typically been classified as M2-polarised, however, comprehensive profiling indicates that the more appropriate classification would be that of a non-polarized M0 phenotype. Additionally, glioblastoma patients have higher numbers of MDSCs in both the tumour parenchyma and peripheral circulation [59,60]. Conversely, there is poor recruitment of T-cells into the glioblastoma TME. T-cells that are present, however, appear to be non-specific to tumour antigen, inactive, and immunosuppressed; characterised by increased expression of co-inhibitory receptors (PD-1, TIM-3) known to suppress effector T cells [61,62].

In this study, we sought to identify secreted molecules that are differentially expressed by serum and GBM cancer stem-like cells. Secreted molecules are largely responsible for defining the tumour microenvironment; therefore, comprehensively profiling the glioblastoma secretome provides valuable insight into the molecular regulation of immune recruitment and modulation in the tumour microenvironment. Specifically, delineating the influence of the small, albeit important, population of gCSCs will aid to include these cells in the development of glioblastoma immunotherapy. Here, we find that serum-derived cultures unanimously produce greater amounts of secreted molecules. This was particularly evident for MCP-1, IL8 and IL6 where the secretion from serum-derived GBM cells was at least 5–50-fold higher than the gCSC counterparts. In contrast, IGFBP2 and sVCAM-1 were secreted at higher levels by the gCSC cells. Gene and protein expression analysis indicates that the differential regulation of these molecules unlikely resides at the level of transcriptional regulation, but at the translational level (Figure 2B and Figure 5).

Overexpressed in glioblastoma, IGFBP2 has previously been associated with promoting cell migration and invasion through binding α5β1 integrin, and activating NFκB mediated migratory phenotypes [49]. IGFBP2 is also up-regulated during neural tube development, particularly within Nestin^+^SOX2^+^ neural stem cells (NSC). Mechanistically, IGFBP2 maintains NSC progenitor populations by regulating cell-cycle exit and promoting proliferation. Furthermore, TCGA analysis reveals that IGFBP2 is up-regulated in classical glioblastoma, reputed for high Nestin expression [63]. We have previously shown that our gCSC cultures significantly up-regulate Nestin expression, giving credence to the correlation between increased IGFBP2 expression and an increased stem-like state [64]. The high levels of IGFBP2 secreted by our gCSC cultures may indicate autocrine regulation of stemness maintenance. Recently, IGFBP2 has been implicated in immunosuppression, where patients with high *IGFBP2* expression have significantly higher levels of genes associated with immunosuppression compared with immune activators. Additionally, IGFBP2 expression positively correlates with increased immune infiltration, and with immunosuppressors such as CHI3L1 and VEGFA [65]. Liu et al. (2019) found that IGFBP2 inhibition increased CD8^+^ T cell populations while concurrently decreasing CD163^+^ macrophages [49]. Reduction in the production or neutralisation of IGFBP2 by the glioblastoma cells is therefore an attractive consideration.

**Figure 8:**
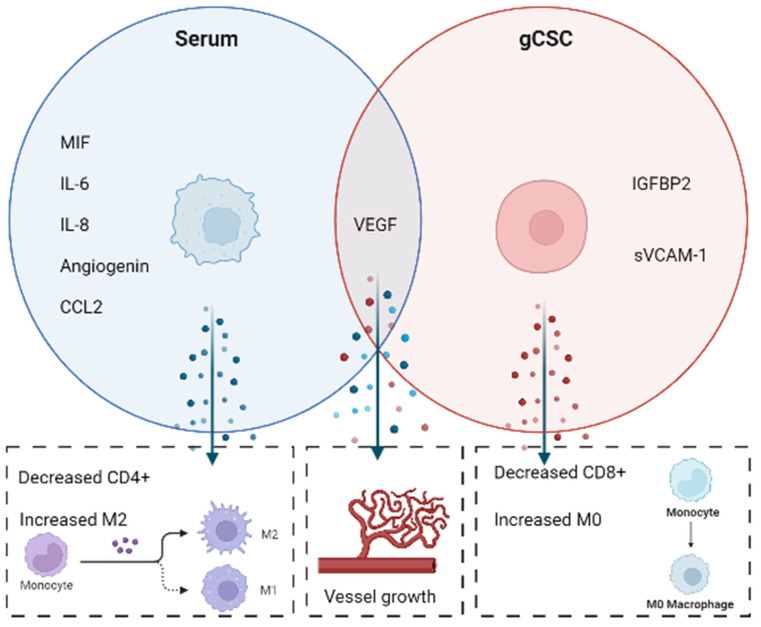
Serum-derived and gCSC cell secreted immune-modulators. Shown are molecules found to be secreted by either serum-derived or gCSC cells, and the associated modulatory role within glioblastoma microenvironments. Figure based on secretome analysis, CIBERSORT, and relevant literature represented in Figure 1 and Table 1.

Next, we used RNA-seqV2 data obtained from The Cancer Genome Atlas to deconvolute the relative proportions of immune subsets within the tumour microenvironment and determine their correlation with tumour cytokine expression (Figure 7). High mRNA levels of IGFBP2 and VEGFA are significantly associated with increased proportions of M0 macrophage phenotypes and decreased M2 phenotypes in the GBM microenvironment. Conversely, molecules enriched in serum-derived cultures (IL-6, MIF, CCL2 and ANG) were associated with increased proportions of M2 macrophages and with decreased proportions of resting CD4^+^ T cells (depicted in Figure 8). Immunosuppressive myeloid cells and M2 macrophages are known to express PD-L1 in glioblastoma patients, both in the TME and peripherally [66,67]. Studies have now demonstrated that myeloid PD-L1 expression is induced by glioblastoma cell IL-6 cytokine secretion acting through STAT3 [68]. Furthermore, IL-6 induces Arg-1 expression on glioma associate macrophages, leading to T cell suppression [69]. A therapeutic rationale arising from this is that anti-IL-6 treatment could be used in combination with checkpoint blockade. Lamano et al. (2019) show that dual anti-IL-6, anti-PD1 therapy increased long-term survival in GL261 tumour-bearing mice [68].

Modulation of the secretory environment in glioblastoma presents (depicted in Figure 8) an inviting avenue of potential therapeutics. Cytokine-based therapeutics are increasingly being used in conjunction with checkpoint blockade and adoptive T cell therapies with clinical success in melanoma, colorectal cancer and renal cell cancer [70]. The rationale for using cytokines in combination with other immunotherapies is to shift the tumour microenvironment towards a state that is receptive to the immunotherapy being used. This approach may be especially effective for oncolytic viral (OV) therapies. As of 2018, 9 of the 43 active clinical trials investigating oncolytic viral therapies were being carried out on glioblastomas and other [71]. The second generation oncolytic herpes simplex virus (HSV), M032, has been developed as dual acting therapy; first to kill tumour cells through oncolytic replication and second to promote the synthesis of IL-12 by tumour cells. By acting as a gene therapy vector, the genetically engineered HSV, manipulates the GBM immune microenvironment to promote increased immune responses against oncolysis-resistant cells [72]. The phase I trial has shown promise in vitro and in vivo and is currently undergoing safety and tolerability testing [71].

Our data here characterises the secretome of both serum-derived and glioblastoma cancer stem-like cells, demonstrating the vast differences between these two cellular populations. Furthermore, we have emphasised the importance of exploring the immunosuppressive roles of molecules such as IGFBP2. Identifying novel immune modulators will provide new therapeutic approaches that could be used to complement current immunotherapies.

## 4. Material and Method

### 4.1. Cell Culture

*New Zealand glioblastoma cell lines.* NZB11, NZB12, NZB13, NZB14, NZB15 and NZB19 primary cell lines provided in collaboration with the Auckland Cancer Society Research Centre were cultured as previously described to establish serum/differentiated and GBM cancer stem-like cells [64]. Serum differentiated cells are large, heterogenous cultures that express markers associated with differentiated astroglial cell types (GFAP, βIII tubulin and Neuronal-N), and fail to form clonal spheres. Contrary, GBM cancer stem-like cells form homogenous cultures that express markers associated with stem cells such as nestin, CD49F, CD44 and A2B5. Furthermore, the stem-like cells form clonal spheres that retain self-renewal properties over multiple generations. See [64] for exhaustive phenotyping details.

*Adherent GBM Cancer Stem-Like Cells (gCSC).* NZB11, NZB12, NZB13, NZB14, NZB15 and NZB19 cell lines were cultured in flasks coated with a solution of 10 µg/mL laminin (ThermoFisher, Waltham, MA, USA). gCSC culture medium consisted of Dulbecco’s Modified Eagle Medium/F12 (DMEM/F12) (ThermoFisher, Waltham, MA, USA) supplemented with 0.5 × B-27 minus vitamin A (ThermoFisher, Waltham, MA, USA), 0.5 × N2 (ThermoFisher, Waltham, MA, USA), 20 ng/mL bFGF (Peprotech, Cranbury, NJ, USA) and 20 ng/mL EGF (Novus Biologicals, Centennial, CO, USA), herein referred to as gCSC cultures. Cultures were maintained at 37 °C, 5% O_2_, 5% CO_2_.

*Serum cultures.* Serum-supplemented cells were cultured in alpha Minimal Essential Medium (αMEM) (ThermoFisher, Waltham, MA, USA) supplemented with 5% FBS (Moregate, Hamilton, New Zealand) and 1 × insulin-transferrin-selenium (ITS) (Sigma-Aldrich, Auckland, New Zealand). Cultures were maintained at 37 °C, 5% O_2_, 5% CO_2_.

### 4.2. NanoString Analysis

Nanostring gene analysis used a custom designed codeset panel containing probes for 40 cytokines, chemokines and immune regulators, and four housekeeping genes (see Table 2). RNA from cell pellets was isolated using RNAqueous TM – Micro Total RNA Isolation Kit (Cat. #AM1931). RNA quality and quantity were determined using NanoDrop TM and Agilent RNA Screentape ^®^. RNA was analysed using the nCounter platform and output data was analysed using nSolver 4.0 advanced analysis (nanoString, Seattle, WA, USA).

Table 2. Details of the nanoString custom designed code-set panel containing probes for 40 cytokines, chemokines and immune modulators, and four housekeeping genes. The gene name, along with the NCBI accession number and target gene sequence is shown.

### 4.3. Cytokine Assays

NZB11, NZB19, NZB12 and NZB13 cells were seeded in 24-well plates using either serum-supplemented or gCSC medium (80,000 cells in one mL) and cultured for 48 h. Conditioned medium was centrifuged at 300× *g* for 5 min. For Proteome Profiler analysis, conditioned medium was added to Human XL Cytokine Arrays (ThermoFisher, Cat. N^o^ ARY022B; Waltham, MA, USA) at a 1 in 3 dilution and assayed according to the manufacturer’s protocol. Blots were imaged by chemiluminescence for 5 min, with multiple exposure times 10 s apart using a Biorad Chemidoc imager. ImageJ software (Wayne Rasband NIH, Bethesda, MA, USA) was used to define each spot according to the array coordinates and analyse pixel intensity. For semi-quantification, background intensity was subtracted, and each spot was normalised to the reference spot intensity. All analyses were performed on exposures immediately prior to reference spot saturation. For multiplex Cytometric Bead Array (CBA, BD Biosciences, Piscataway, NJ, USA), media samples were stored at −80 °C. The concentration of cytokines and chemokines was determined according to the manufacturer’s protocol (BD^TM^ Cytometric Bead Array Kits). An Accuri C6 flow cytometer (BD Biosciences, Piscataway, NJ, USA) was used to measure analyte concentrations, and FCAP array software (v3.0.1, BD Biosciences, Piscataway, NJ, USA) was used to process data. Soluble proteins (serpine-E1, IGFBP2 and CHI3L1), which could not be detected using CBA kits, were quantified using Luminex analysis following storage at −80 °C (R&D Systems Human Luminex Assay (6-plex)).

### 4.4. TIMER 2.0 and CIBERSORT

RNAseqV2 data normalised using RSEM (RNA-seq by Expectation-Maximization) from 160 glioblastoma patients (TCGA, PanCancer Atlas) was downloaded from cBioPortal [73,74]. The data was sorted from highest expressing samples to lowest expressing samples for *IL6*, *IGFBP2*, *MIF*, *ANG*, *CHI3L1*, *SERPINE1*, *VEGFA* and *IL8* and separated into quartiles containing 40 samples each. Quartile 1 (highest expression) and quartile 4 (lowest expression) were uploaded into TIMER 2.0 estimation software (see http://timer.cistrome.org/, accessed on 1 July 2022) set to cancer type glioblastoma [75,76,77]. CIBERSORT data of 22 deconvoluted immune subtypes was obtained to generate violin plots comparing immune cell fractions between quartile 1 and quartile 4 samples [78].

### 4.5. Gene Set Enrichment Analysis (GSEA)

Gene set enrichment analysis was performed to evaluate RNA-seq data from 160 glioblastoma patients (TCGA, PanCancer Atlas) downloaded from cBioPortal [73,74]. The data was phenotypically separated into highest (Q1) expressing samples to lowest (Q4) expressing samples for *IL6*, *IGFBP2*, *MIF*, *ANG*, *CHI3L1*, *SERPINE1*, *VEGFA* and *IL8*. MSigDB software provided pre-defined gene sets corresponding to the gene ontology (GO) for Macrophage Activation, NK Activation, T cell Activation, Macrophage Migration, T cell Migration and T cell Proliferation.

### 4.6. Statistics

Nanostring, Cytometric Bead Array and Luminex data were analysed with unpaired students T-tests statistical comparisons. GraphPad v.7 (Dotmatics, San Diego, CA, USA) was used to generate statistical tests. Normality for CIBERSORT data was determined using Kolmogorov–Smirnov test with Lilliefors Significance Correction. Mann–Whitney *U* was used for CIBERSORT statistical comparisons. IBM SPSS Statistics v.27 (IBM, New York, USA) was used to generate statistical tests. *p*-value = 0.05 (*), 0.01 (**), 0.001 (***), 0.0001(****).

## Figures and Tables

**Table 1:** Citations of depicted secreted proteins presented in Figure 1.

MIF	[13,14,15,16,17]
CCL2	[18,19,20,21]
CXCL8	[22,23,24,25,26,27]
CX3CL1	[28,29]
CXCL2	[16,30,31,32]
CXCL5	[31,33,34]
CXCL3	[35,36]
CXCL1	[30,37,38]
CHI3L1	[39,40]
Serpin E1	[41,42]
VEGFA	[43,44,45,46,47]
IGFBP2	[48,49]
sICAM1	[50]
IL-6	[51,52]

**Table 2:** NanoString Probes.

Gene	Accession	Target Sequence
**Housekeepers**		
**MRPS5**	NM_031902.3	ATCCCTACGCCAGCTTGAGCCGTGCACTGCAGACACAATGCTGTATTTCTTCTCCCAGTCACCTGATGAGCCAGCAGTATAGACCATATAGTTTCTTCAC
**PCNA**	NM_002592.2	GGTGTTGGAGGCACTCAAGGACCTCATCAACGAGGCCTGCTGGGATATTAGCTCCAGCGGTGTAAACCTGCAGAGCATGGACTCGTCCCACGTCTCTTTG
**PPIA**	NM_021130.3	TCTATGGGGAGAAATTTGAAGATGAGAACTTCATCCTAAAGCATACGGGTCCTGGCATCTTGTCCATGGCAAATGCTGGACCCAACACAAATGGTTCCCA
**TBP**	NM_001172085.1	ACAGTGAATCTTGGTTGTAAACTTGACCTAAAGACCATTGCACTTCGTGCCCGAAACGCCGAATATAATCCCAAGCGGTTTGCTGCGGTAATCATGAGGA
**Cytokines**		
**IL10**	NM_000572.2	AAGGATCAGCTGGACAACTTGTTGTTAAAGGAGTCCTTGCTGGAGGACTTTAAGGGTTACCTGGGTTGCCAAGCCTTGTCTGAGATGATCCAGTTTTACC
**IL1A**	NM_000575.3	ACTCCATGAAGGCTGCATGGATCAATCTGTGTCTCTGAGTATCTCTGAAACCTCTAAAACATCCAAGCTTACCTTCAAGGAGAGCATGGTGGTAGTAGCA
**IL2**	NM_000586.2	AGGATGCAACTCCTGTCTTGCATTGCACTAAGTCTTGCACTTGTCACAAACAGTGCACCTACTTCAAGTTCTACAAAGAAAACACAGCTACAACTGGAGC
**CSF3**	NM_000759.3	CCTGCATTTCTGAGTTTCATTCTCCTGCCTGTAGCAGTGAGAAAAAGCTCCTGTCCTCCCATCCCCTGGACTGGGAGGTAGATAGGTAAATACCAAGTAT
**IL22**	NM_020525.4	CTATCTGATGAAGCAGGTGCTGAACTTCACCCTTGAAGAAGTGCTGTTCCCTCAATCTGATAGGTTCCAGCCTTATATGCAGGAGGTGGTGCCCTTCCTG
**IL12A**	NM_000882.2	CTTTCTAGATCAAAACATGCTGGCAGTTATTGATGAGCTGATGCAGGCCCTGAATTTCAACAGTGAGACTGTGCCACAAAAATCCTCCCTTGAAGAACCG
**IL6**	NM_000600.3	GGCACTGGCAGAAAACAACCTGAACCTTCCAAAGATGGCTGAAAAAGATGGATGCTTCCAATCTGGATTCAATGAGGAGACTTGCCTGGTGAAAATCATC
**IL1B**	NM_000576.2	GGGACCAAAGGCGGCCAGGATATAACTGACTTCACCATGCAATTTGTGTCTTCCTAAAGAGAGCTGTACCCAGAGAGTCCTGTGCTGAATGTGGACTCAA
**IL17A**	NM_002190.2	TACTACAACCGATCCACCTCACCTTGGAATCTCCACCGCAATGAGGACCCTGAGAGATATCCCTCTGTGATCTGGGAGGCAAAGTGCCGCCACTTGGGCT
**Chemokines**		
**CCL1**	NM_002981.1	CCTTCTCCAGATGTTGCTTCTCATTTGCGGAGCAAGAGATTCCCCTGAGGGCAATCCTGTGTTACAGAAATACCAGCTCCATCTGCTCCAATGAGGGCTT
**CCL11**	NM_002986.2	TGGGTGCAGGATTCCATGAAGTATCTGGACCAAAAATCTCCAACTCCAAAGCCATAAATAATCACCATTTTTGAAACCAAACCAGAGCCTGAGTGTTGCC
**CCL17**	NM_002987.2	GCCTGGAGTACTTCAAGGGAGCCATTCCCCTTAGAAAGCTGAAGACGTGGTACCAGACATCTGAGGACTGCTCCAGGGATGCCATCGTTTTTGTAACTGT
**CCL19**	NM_006274.2	GACCTCAGCCAAGATGAAGCGCCGCAGCAGTTAACCTATGACCGTGCAGAGGGAGCCCGGAGTCCGAGTCAAGCATTGTGAATTATTACCTAACCTGGGG
**CCL2**	NM_002982.3	CATTCCCCAAGGGCTCGCTCAGCCAGATGCAATCAATGCCCCAGTCACCTGCTGTTATAACTTCACCAATAGGAAGATCTCAGTGCAGAGGCTCGCGAGC
**CCL23**	NM_005064.5	TGAGAATGCTGAAGCTGGACACACGGATCAAGACCAGGAAGAATTGAACTTGTCAAGGTGAAGGGACACAAGTTGCCAGCCACCAACTTTCTTGCCTCAA
**CCL3**	NM_002983.2	CTGTGTAGGCAGTCATGGCACCAAAGCCACCAGACTGACAAATGTGTATCGGATGCTTTTGTTCAGGGCTGTGATCGGCCTGGGGAAATAATAAAGATGC
**CCL5**	NM_002985.2	AGTGTGTGCCAACCCAGAGAAGAAATGGGTTCGGGAGTACATCAACTCTTTGGAGATGAGCTAGGATGGAGAGTCCTTGAACCTGAACTTACACAAATTT
**CX3CL1**	NM_002996.3	CCCCGGAGCTGTGGTAGTAATTCATATGTCCTGGTGCCCGTGTGAACTCCTCTGGCCTGTGTCTAGTTGTTTGATTCAGACAGCTGCCTGGGATCCCTCA
**CXCL1**	NM_001511.1	TATGTTAATATTTCTGAGGAGCCTGCAACATGCCAGCCACTGTGATAGAGGCTGGCGGATCCAAGCAAATGGCCAATGAGATCATTGTGAAGGCAGGGGA
**CXCL2**	NM_002089.3	ATCACATGTCAGCCACTGTGATAGAGGCTGAGGAATCCAAGAAAATGGCCAGTGAGATCAATGTGACGGCAGGGAAATGTATGTGTGTCTATTTTGTAAC
**CXCL3**	NM_002090.2	TCCCTGCCCTTACCAGAGCTGAAAATGAAAAAGAGAACAGCAGCTTTCTAGGGACAGCTGGAAAGGACTTAATGTGTTTGACTATTTCTTACGAGGGTTC
**CXCL5**	NM_002994.3	AGAGAGCTGCGTTGCGTTTGTTTACAGACCACGCAAGGAGTTCATCCCAAAATGATCAGTAATCTGCAAGTGTTCGCCATAGGCCCACAGTGCTCCAAGG
**CXCL8**	NM_000584.2	ACAGCAGAGCACACAAGCTTCTAGGACAAGAGCCAGGAAGAAACCACCGGAAGGAACCATCTCACTGTGTGTAAACATGACTTCCAAGCTGGCCGTGGCT
**CXCL9**	NM_002416.1	CACCATCTCCCATGAAGAAAGGGAACGGTGAAGTACTAAGCGCTAGAGGAAGCAGCCAAGTCGGTTAGTGGAAGCATGATTGGTGCCCAGTTAGCCTCTG
**MIF**	NM_002415.1	TCCTACAGCAAGCTGCTGTGCGGCCTGCTGGCCGAGCGCCTGCGCATCAGCCCGGACAGGGTCTACATCAACTATTACGACATGAACGCGGCCAATGTGG
**Immune modulators**		
**ANG**	NM_001145.4	AGTACCGAGCCACAGCGGGGTTCAGAAACGTTGTTGTTGCTTGTGAAAATGGCTTACCTGTCCACTTGGATCAGTCAATTTTCCGTCGTCCGTAACCAGC
**CHI3L1**	NM_001276.2	GGTCTCAAAGATTTTCCAAGATAGCCTCCAACACCCAGAGTCGCCGGACTTTCATCAAGTCAGTACCGCCATTTCTGCGCACCCATGGCTTTGATGGGCT
**CST3**	NM_000099.2	CCCTTCCATGACCAGCCACATCTGAAAAGGAAAGCATTCTGCTCTTTCCAGATCTACGCTGTGCCTTGGCAGGGCACAATGACCTTGTCGAAATCCACCT
**ENG**	NM_001114753.1	GTCCTTGATCCAGACAAAGTGTGCCGACGACGCCATGACCCTGGTACTAAAGAAAGAGCTTGTTGCGCATTTGAAGTGCACCATCACGGGCCTGACCTTC
**FASLG**	NM_000639.1	TCCATGCCTCTGGAATGGGAAGACACCTATGGAATTGTCCTGCTTTCTGGAGTGAAGTATAAGAAGGGTGGCCTTGTGATCAATGAAACTGGGCTGTACT
**ICAM1**	NM_000201.2	AAATACTGAAACTTGCTGCCTATTGGGTATGCTGAGGCCCCACAGACTTACAGAAGAAGTGGCCCTCCATAGACATGTGTAGCATCAAAACACAAAGGCC
**IDO1**	NM_002164.5	ATCACCATGGCATATGTGTGGGGCAAAGGTCATGGAGATGTCCGTAAGGTCTTGCCAAGAAATATTGCTGTTCCTTACTGCCAACTCTCCAAGAAACTGG
**IFNG**	NM_000619.2	ATACTATCCAGTTACTGCCGGTTTGAAAATATGCCTGCAATCTGAGCCAGTGCTTTAATGGCATGTCAGACAGAACTTGAATGTGTCAGGTGACCCTGAT
**IGFBP2**	NM_000597.2	TCGGGTATGAAGGAGCTGGCCGTGTTCCGGGAGAAGGTCACTGAGCAGCACCGGCAGATGGGCAAGGGTGGCAAGCATCACCTTGGCCTGGAGGAGCCCA
**IGFBP3**	NM_000598.4	TATCAAAATATTCAGAGACTCGAGCACAGCACCCAGACTTCATGCGCCCGTGGAATGCTCACCACATGTTGGTCGAAGCGGCCGACCACTGACTTTGTGA
**SERPINE1**	NM_000602.2	TGTGTTCAATAGATTTAGGAGCAGAAATGCAAGGGGCTGCATGACCTACCAGGACAGAACTTTCCCCAATTACAGGGTGACTCACAGCCGCATTGGTGAC
**SPP1**	NM_000582.2	CGCCTTCTGATTGGGACAGCCGTGGGAAGGACAGTTATGAAACGAGTCAGCTGGATGACCAGAGTGCTGAAACCCACAGCCACAAGCAGTCCAGATTATA
**TGFB1**	NM_000660.3	TATATGTTCTTCAACACATCAGAGCTCCGAGAAGCGGTACCTGAACCCGTGTTGCTCTCCCGGGCAGAGCTGCGTCTGCTGAGGCTCAAGTTAAAAGTGG
**VCAM1**	NM_001078.3	CAGACTTCCCTGAATGTATTGAACTTGGAAAGAAATGCCCATCTATGTCCCTTGCTGTGAGCAAGAAGTCAAAGTAAAACTTGCTGCCTGAAGAACAGTA
**VEGFA**	NM_001025366.1	GAGTCCAACATCACCATGCAGATTATGCGGATCAAACCTCACCAAGGCCAGCACATAGGAGAGATGAGCTTCCTACAGCACAACAAATGTGAATGCAGAC

## Data Availability

All data for this study is held by the lead author E Scott Graham.

## Supplementary Materials

The following supporting information can be downloaded at: https://www.mdpi.com/article/10.3390/ijms232214164/s1.
