# Supplementary material for: Comprehensive Assessment of Secreted Immuno-Modulatory Cytokines by Serum-Differentiated and Stem-like Glioblastoma Cells Reveals Distinct Differences between Glioblastoma Phenotypes
Source: Int J Mol Sci. 2022 Nov 16;23(22):14164. doi: 10.3390/ijms232214164 (PMC9692434; doi:10.3390/ijms232214164)
Supplement: Supplementary file 1 [file ijms-23-14164-s001.zip › ijms-1903714-supplementary.pdf]

Comprehensive assessment of secreted immuno-modulatory cytokines by serum-differentiated and stem-like glioblastoma cells reveals distinct differences between glioblastoma phenotypes

Laverne D Robilliard, Jane Yu, Akshata Anchan, Graeme Finlay, Catherine E Angel, and E Scott Graham.

Supplemental Figures

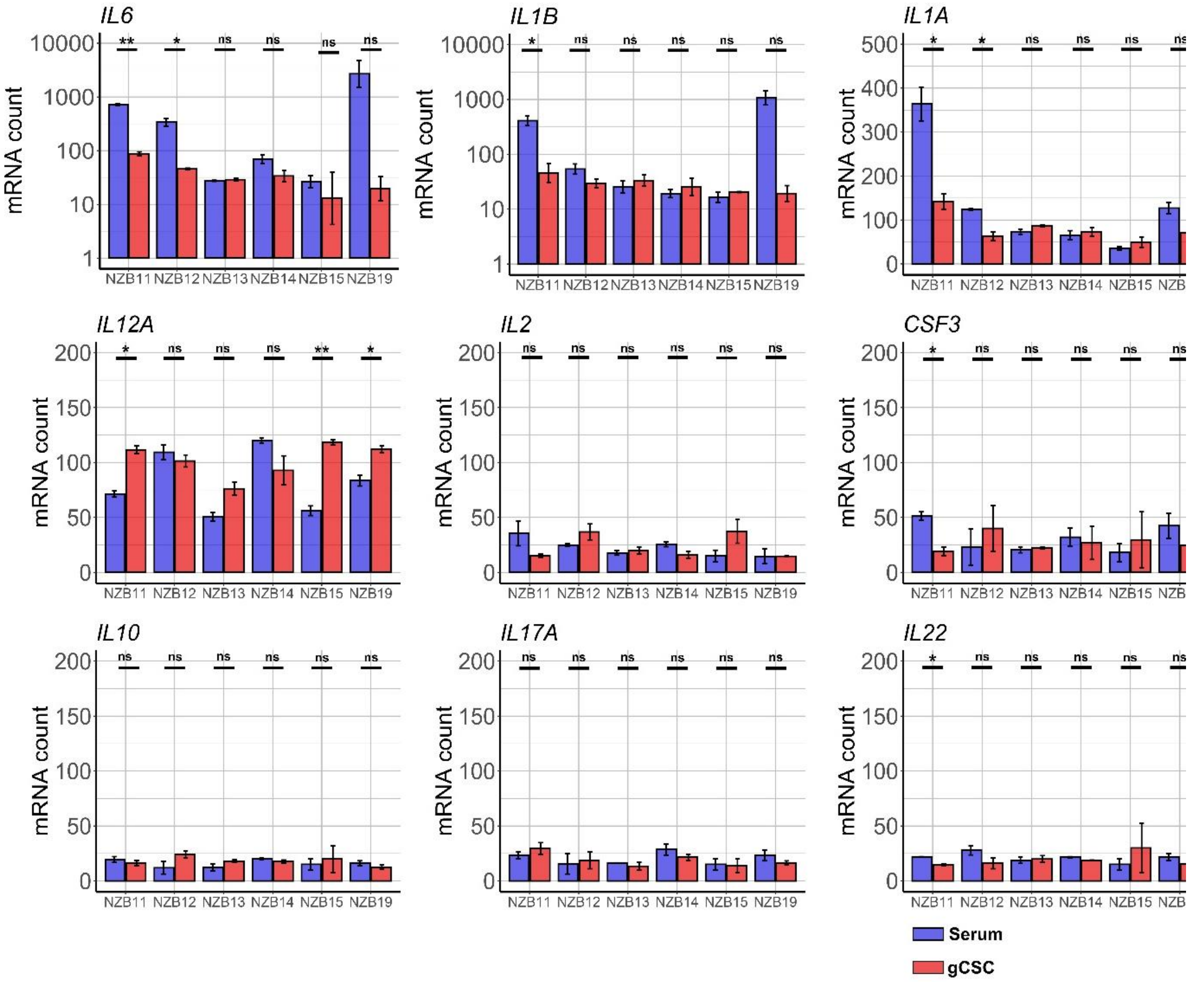

Figure S 1 Cytokine gene expression by glioblastoma cells. Nanostring analysis of absolute mRNA count in NZB11, NZB19, NZB12, NZB13, NZB14 and NZB15 GBM lines. The results of two independent experiments are shown. Unpaired students *t*-test analysis was carried out. *P*-value = 0.05 (*), 0.01 (**), 0.001 (***), 0.0001(****).

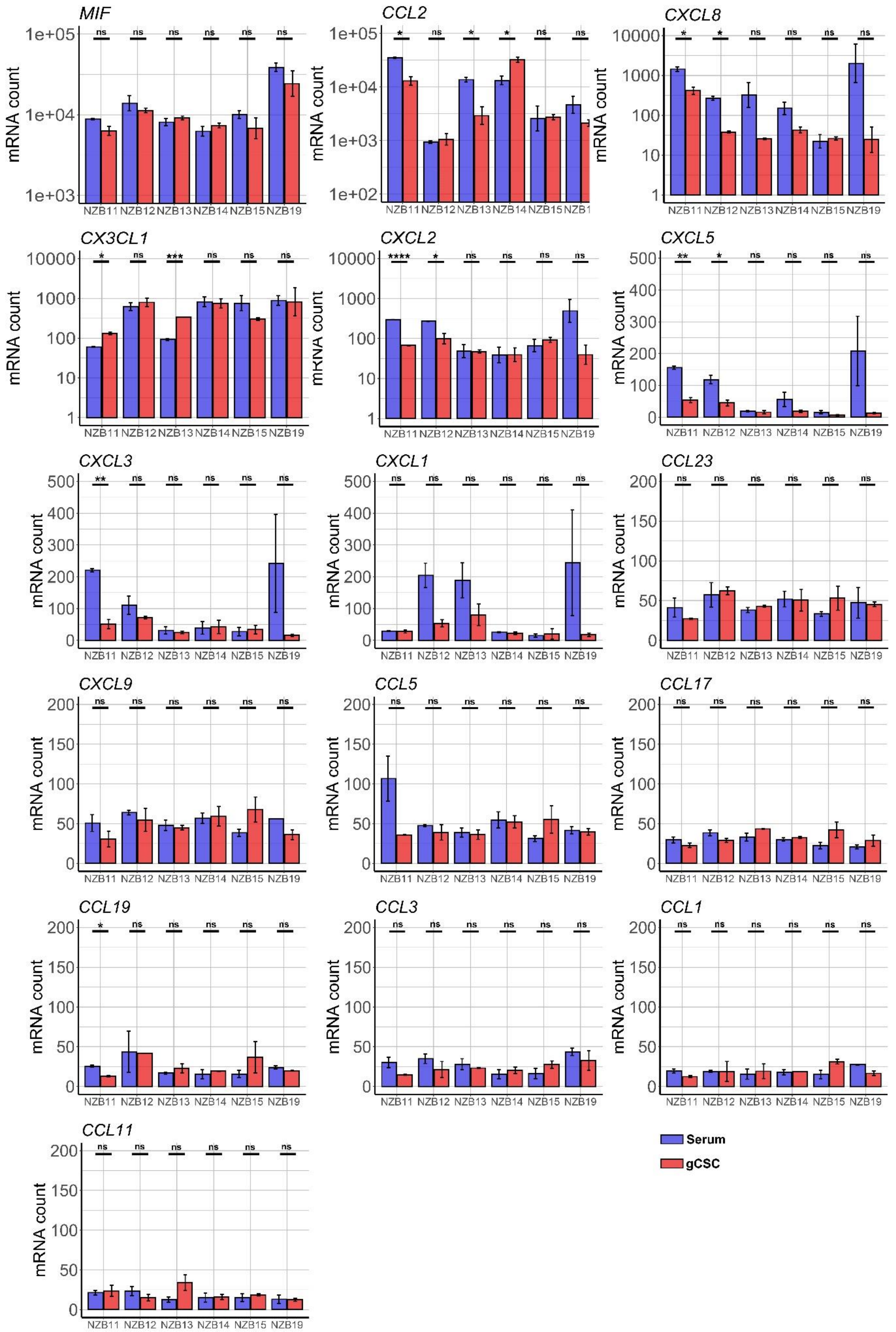

Figure S 2 Chemokine gene expression by glioblastoma cells. Nanostring analysis of absolute mRNA count in NZB11, NZB19, NZB12, NZB13, NZB14 and NZB15 GBM lines. The results of two

independent experiments are shown. Unpaired students *t*-test analysis was carried out. *P*-value = 0.05 (*), 0.01 (**), 0.001 (***), 0.0001(****).

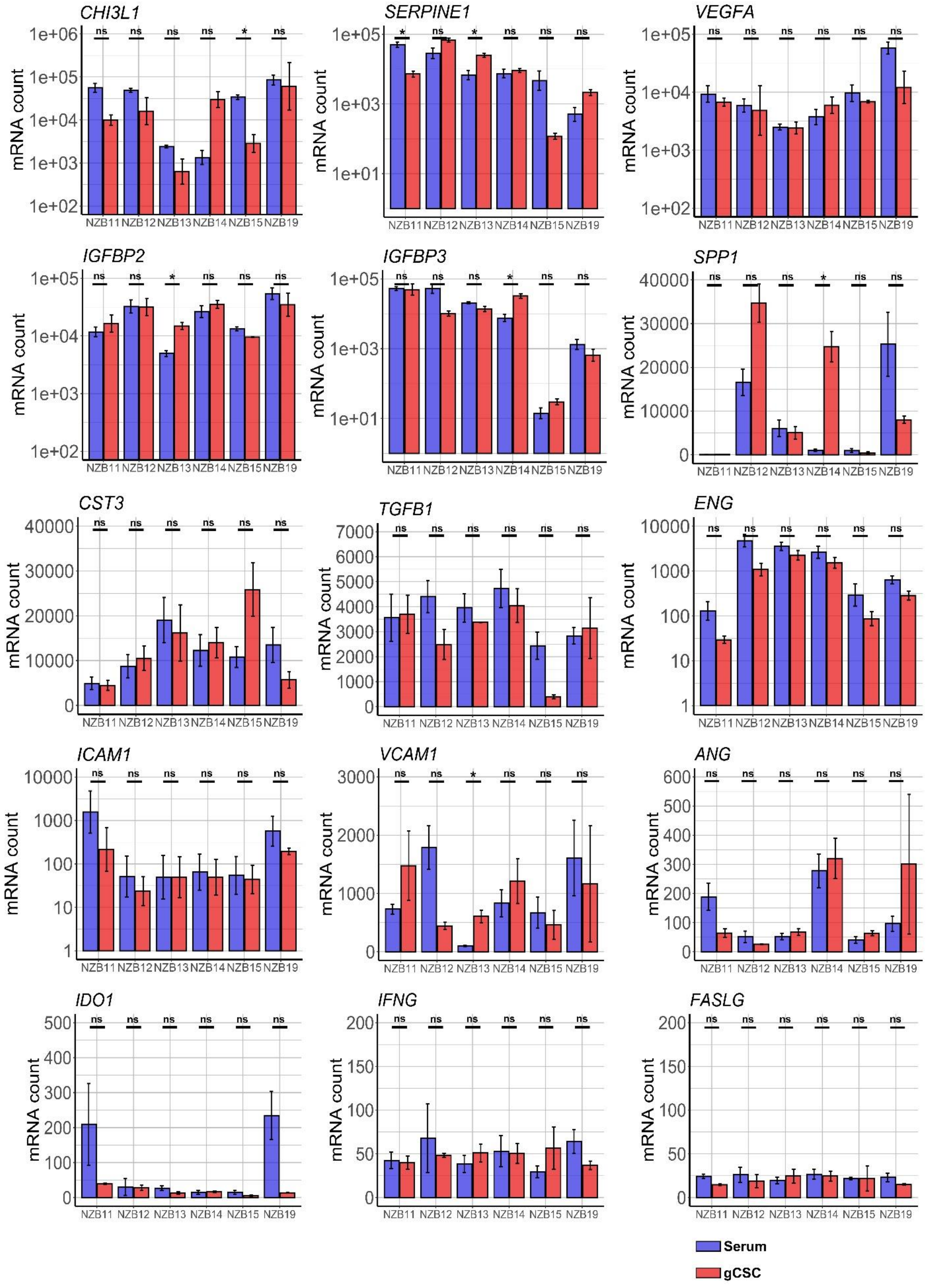

Figure S 3 Non-classical immune modulator gene expression by glioblastoma cells. Nanostring analysis of absolute mRNA count in NZB11, NZB19, NZB12, NZB13, NZB14 and NZB15 GBM lines. The results of two independent experiments are shown. Unpaired students *t*-test analysis was carried out. *P*-value = 0.05 (*), 0.01 (**), 0.001 (***), 0.0001(****).

A

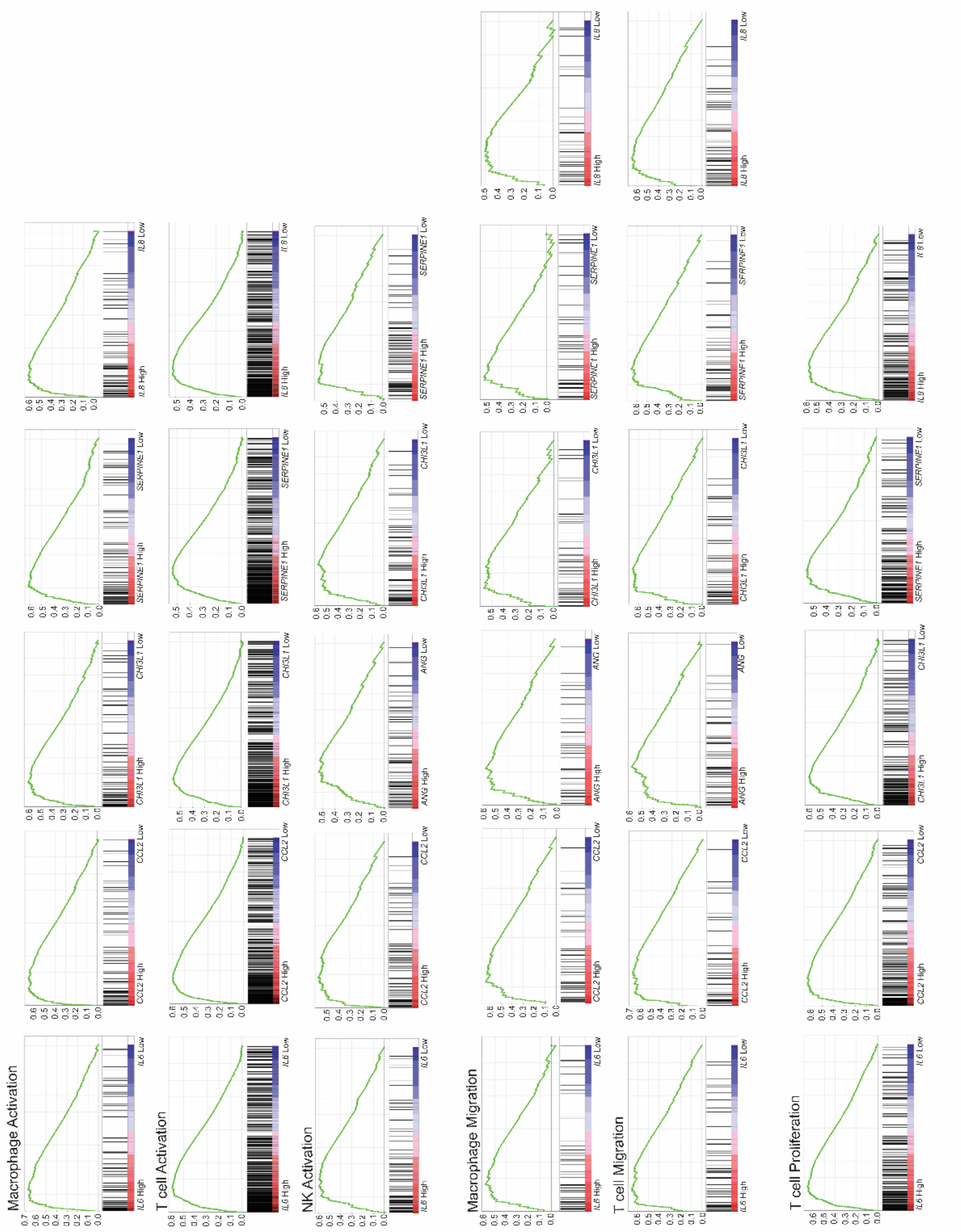

B

	IL6			*IGFBP2*			*MIF*			*CCL2*			*ANG*			*CHI3L1*			*SERPINE1*			*VEGFA*			*IL8*		
	NES	p-val	FDR q-val	NES	p-val	FDR q-val	NES	p-val	FDR q-val	NES	p-val	FDR q-val	NES	p-val	FDR q-val	NES	p-val	FDR q-val	NES	p-val	FDR q-val	NES	p-val	FDR q-val	NES	p-val	FDR q-val
Macrophage Activation	2.06	***	0.00	-1.08	ns	0.67	0.87	ns	0.56	2.08	****	0.00	1.65	ns	0.03	1.88	**	0.01	1.87	**	0.03	-0.84	ns	0.63	1.92	***	0.01
T cell Activation	2.09	****	0.00	-0.66	ns	0.83	0.74	ns	0.94	2.05	****	0.00	1.62	ns	0.10	2.08	****	0.01	1.87	*	0.03	-0.99	ns	0.53	1.85	**	0.03
NK Activation	1.92	****	0.00	-0.79	ns	0.73	0.78	ns	1.00	1.87	**	0.01	1.66	*	0.09	1.88	**	0.01	1.80	**	0.05	-1.13	ns	0.32	1.51	ns	0.08
					ns			ns															ns				
Macrophage Migration	1.79	**	0.01	-0.89	ns	0.73	1.06	ns	1.00	1.98	**	0.00	1.82	**	0.02	1.71	**	0.02	1.65	*	0.03	-1.20	ns	0.29	1.58	*	0.05
T cell Migration	1.88	*	0.01	-0.83	ns	0.90	0.71	ns	1.00	1.96	****	0.00	1.68	*	0.10	1.91	**	0.01	1.87	**	0.04	-0.97	ns	0.52	1.90	***	0.03
					ns			ns															ns				
T cell Proliferation	2.14	****	0.00	-0.89	ns	1.00	0.98	ns	0.85	2.05	****	0.00	1.66	ns	0.10	2.03	**	0.01	1.85	*	0.02	-1.26	ns	0.81	1.89	**	0.02

Figure S 4 Gene set enrichment analysis (GSEA). Gene set enrichment analysis was performed on phenotypically high (Q1) (n=40) and low (Q4) (n=40) glioblastoma samples from a publicly available GBM RNA-seq dataset (n=160). The GSEA algorithm shows the concordant differences between high and low expressing GBM samples for *IL6*, *IGFBP2*, *MIF*, *CCL2*, *ANG*, *CHI3L1*, *SERPINE1*, *VEGF*, and *IL8*. The defined gene sets correspond to the Gene Ontology (GO) terms for Macrophage Activation, T cell Activation, NK Activation, Macrophage Migration, T cell Migration, and T cell proliferation. A) Statistically significant enrichment plots between Q1 and Q4 phenotypes across six GO term. B) Table of all normalised enrichment scores (NES) with corresponding *p*-values and false discovery rate *q*-values for six GO terms. Positive NES are positive correlations with Q1, and negative NES are negative correlations for Q1. FDR cut-off of 0.25 and *p*-values less than 0.05 were used to define significance. *P*-value = 0.05 (*), 0.01 (**), 0.001 (***), 0.0001 (****).
